# Probiotics as a New Regulator for Bone Health: A Systematic Review and Meta-Analysis

**DOI:** 10.1155/2021/3582989

**Published:** 2021-08-02

**Authors:** Hanieh Malmir, Hanieh-Sadat Ejtahed, Ahmad-Reza Soroush, Amir Mohammad Mortazavian, Noushin Fahimfar, Afshin Ostovar, Ahmad Esmaillzadeh, Bagher Larijani, Shirin Hasani-Ranjbar

**Affiliations:** ^1^Obesity and Eating Habits Research Center, Endocrinology and Metabolism Clinical Sciences Institute, Tehran University of Medical Sciences, Tehran, Iran; ^2^Department of Community Nutrition, School of Nutritional Sciences and Dietetics, Tehran University of Medical Sciences, Tehran, Iran; ^3^Endocrinology and Metabolism Research Center, Endocrinology and Metabolism Clinical Sciences Institute, Tehran University of Medical Sciences, Tehran, Iran; ^4^Department of Food Science and Technology, Faculty of Nutrition Sciences and Food Technology, Shahid Beheshti University of Medical Sciences, Tehran, Iran; ^5^Osteoporosis Research Center, Endocrinology and Metabolism Clinical Sciences Institute, Tehran University of Medical Sciences, Tehran, Iran

## Abstract

Despite the proposed role of the gut microbiota-bone axis, findings on the association between probiotic consumption and bone health are conflicting. This systematic review aimed to assess the effect of probiotic consumption on bone health parameters. A systematic literature search of relevant reports published in PubMed/Medline, Web of Science, SCOPUS, EMBASE, and Google scholar before December 2020 was conducted. All clinical trials or experimental studies, which examined the relationship between probiotic consumption and bone health parameters, were included. No limitation was applied during the search. After screening articles based on inclusion criteria, 44 studies remained. In clinical trials, probiotic consumption affects bone health parameters such as serum calcium levels (3.82; 95% CI: 1.05, 6.59 mmol/l), urinary calcium levels (4.85; 95% CI: 1.16, 8.53 mmol/l), and parathyroid hormone (PTH) levels (−5.53; 95% CI: −9.83, −0.86 ng/l). In most studies, *Lactobacillus* species such as *L. helveticus, L. reuteri*, and *L. casei* were consumed and women aged 50 years or older were assessed. Spinal and total hip bone mineral density (BMD) was not affected significantly by probiotic consumption. In 37 animal experiments, probiotic or symbiotic feeding mostly had effects on bone health parameters. Some strains of *Bifidobacterium* and *Lactobacillus* including *L*. *reuteri, L. casei*, *L. paracasei, L. bulgaricus*, and *L. acidophilus* have indicated beneficial effects on bone health parameters. In conclusion, this systematic review and meta-analysis indicate that probiotic supplementation might improve bone health. Further studies are needed to decide on the best probiotic species and appropriate dosages.

## 1. Introduction

Bone health is critically important to the overall health and quality of life and depends on the balance of bone resorption and bone formation [1]. Low bone mineral density (BMD) value is an indicator of osteoporosis or fracture and one of the major public health problems [2]. Significant disability, increased dependency, reduced quality of life, and increased economic burden to the health care system are the most consequences of reduced BMD [3–5]. BMD is affected by various factors, including gender, age, family history, fracture history, alcohol consumption, tobacco smoking, taking some medicines, bone diseases, and lack of physical activity [6–11]. Moreover, previous studies indicated that hormone status, immune cells, and the gastrointestinal system can also regulate bone balances and health [12]. The gastrointestinal tract has a key role in the absorption of calcium, phosphorous, and magnesium as contributors to bone mineralization and produces endocrine factors that signal to bone cells, such as Incretin and serotonin. Furthermore, the gut microbiota has been proposed as a regulator of bone health [13].

The human gut microbiota comprises over 1000 distinct microbial species [14]. According to Codex Alimentarius, probiotics are live microorganisms that provide health benefits, generally by improving or restoring the gut microbial flora [15]. Although, they are recommended to be defined as the viable or unviable microbial cell (vegetative or spore; intact or ruptured) that is potentially healthful to the host [16]. Disturbed gut microbiota composition contributes to many human chronic diseases, such as obesity, metabolic diseases, malnutrition, neurological disorders, cardiovascular disease, and cancer [17]. Also, bone mass and bone quality are affected by gut microbiota [18]. Previous studies have indicated the relation between bone health and probiotics. For example, in Takimoto et al. study, oral probiotic supplementation stimulates the diversity of gut microbiota as well as bone growth and density [19]. Although most of the previous publications indicated beneficial effects of probiotics on bone health [19–21], some others had not found any association between probiotic consumption and bone health status [22, 23]. In Sergeev et al.'s study, three-month supplementation by a probiotic capsule had no effects on the bone mineral content in overweight and obese adults [22]. Despite these controversies, no publication summarized findings from previous publications in this field. Although bone health and diseases have a significant role on affected individuals, they also affect the population [24]. Osteoporosis has increased economic burden to health care system, as well as loss of income to the employee, loss of productivity to the employer, costs to a country's social welfare system including unemployment and disability pay, health insurance payments, and rises in insurance premiums [25]. Due to the deep effects of osteoporosis and fractures on society and increasing prevalence of osteoporosis [26], it seems that preventing studies are more important. This study, therefore, aimed to comprehensively review previous animal studies and clinical trials about the effect of probiotic consumption on bone health status.

## 2. Materials and Methods

This systematic review was performed according to the Preferred Reporting Items for Systematic Reviews and Meta-Analysis (PRISMA) statement with the aim of assessing the relation between probiotic consumption and bone health parameters and has been recorded in PROSPERO.

### 2.1. Search Strategy

Previous publications on the effect of probiotic consumption and bone health status were selected through searching in PubMed/Medline, ISI Web of Science, SCOPUS, Cochrane, and EMBASE up to December 2020. The following key words were used in this search: probiotics OR synbiotic OR *Lactobacillus* OR *Bifidobacterium* for the intake of probiotics, and osteoporosis OR fracture OR “bone mineral density” OR BMD OR “bone mineral content” OR “alkaline phosphatase” OR osteocalcin OR “procollagen type 1 N-terminal propeptide” OR hydroxyproline OR “NF-κB ligand” for bone health status (Supplemental Table 1). In PubMed, keywords were searched through [tiab] and [MeSH] tags. No limitation was applied during the search. The reference lists of retrieved papers were also examined to avoid missing any published data. Finally, articles in the English language were included.

### 2.2. Inclusion and Exclusion Criteria

Two investigators independently selected the articles through the mentioned search strategy. Publications that fulfilled the following criteria were eligible for inclusion: (1) all studies with clinical trial design or experimental design (animal studies); (2) studies that examined the relationship between probiotic consumption and bone health status parameters; and (3) those that reported quantity findings for probiotic consumption and bone health parameters. We excluded letters, comments, reviews, meta-analyses, ecological, and in vitro studies as well as duplicate studies. Inclusion criteria based on PICOS include the following: Population, adults (for human studies) and other animals for experimental studies; Intervention/Exposure, probiotic consumption; Comparison, consumption and nonconsumption of probiotics; Outcome: bone health parameters; Study design: clinical trial design or experimental design (animal studies).

### 2.3. Data Extraction

For each eligible study, the following information was extracted: first author, year of publication, study design, country, age range, gender, sample size (number of participants in each group), type of intervention, duration of intervention, the dose of probiotic intake in the intervention group, characteristics of the control group, outcome variables, the mean and standard deviation of bone health parameters in the intervention and control groups, and quality score.

### 2.4. Quality Assessment of Studies

The risk of bias for the included studies was evaluated using the Cochrane quality assessment tool for RCTs. Two independent investigators assessed the quality of studies using the following seven criteria: (i) random sequence generation, (ii) allocation concealment, (iii) blinding of participants and personnel, (iv) blinding of outcome assessment, (v) incomplete outcome data, (vi) selective reporting, and (vii) other probable sources of biases. To evaluate the quality of studies, each study was allocated a label (yes, no, or unclear) indicating that it was judged as low risk, high risk, or unknown risk of bias, respectively [27].

All steps of the methods were performed by two investigators independently including searching, article screening, and data extracting, and checking the quality of articles. Disagreements between the two investigators were resolved by discussion and consensus.

### 2.5. Statistical Analysis

Mean differences ± SDs of measures such as chemical bone health parameters and BMD, comparing probiotic consumption to control, were used to calculate the overall effect sizes. When mean differences ± SDs were not reported, we calculated them by considering changes in each parameter throughout the study. In addition, these parameters were reported in different units across the studies. We converted them to the same units. The overall effect size was calculated by using a random effects model, which takes between-study variation into account. Cochran's Q test and *I*2 statistic were used to assess between-study heterogeneity. Sensitivity analysis was used to explore the extent to which inferences might depend on a particular study or group of studies. Publication bias was examined by visual inspection of funnel plots and the application of Egger's and Begg's tests. We used kappa statics to assess the consonant between investigators. All statistical analyses were conducted by using STATA version 14.2 (StataCorp). *P* values <0.05 were considered significant.

## 3. Results

**Figure 1:**
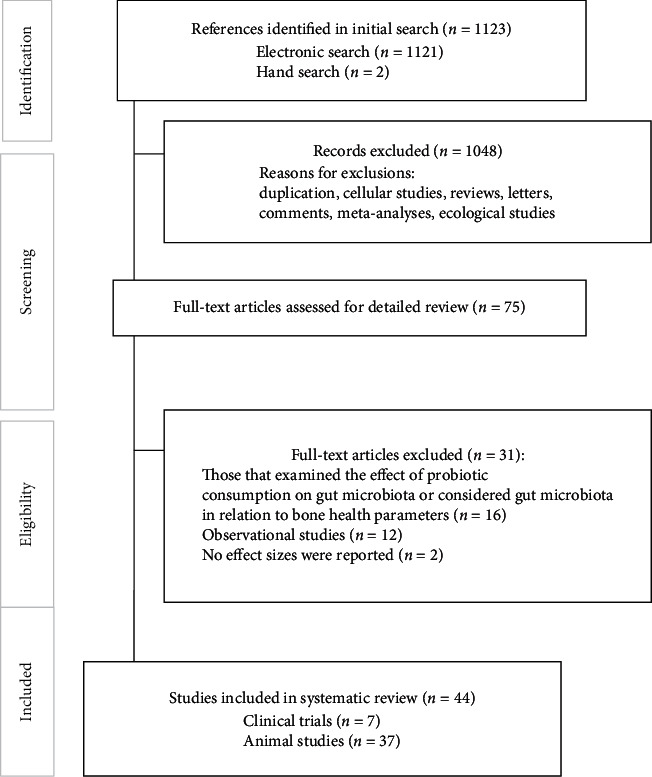
The flow diagram of study selection.

In total, 1123 articles were found in our initial search. After exclusion of duplicate studies and screening nonrelated articles based on title and abstract, 75 articles were remained. We further excluded 31 papers because of the following reasons: (1) those that examined the effect of probiotic consumption on gut microbiota without considering the effects of probiotics on bone health status or assessed the relationship between gut microbiota and bone health parameters without intervention (*n* = 16); (2) publications in which no effect sizes were reported (*n* = 3); and (3) those that had observational design (cohort, case-control, or cross-sectional design) (*n* = 12). After these exclusions, 44 papers remained for the current systematic review (Figure 1). Two investigators independently selected the articles through the mentioned search strategy and they had high agreement (0.90). The disagreement between the two investigators was resolved by the opinion of the third one.

### 3.1. Animal Studies

Characteristics of 37 animal studies on the effects of probiotics on bone parameters are presented in Table 1. These investigations are published between 2004 and 2020. Most of them were performed on rats except for four studies performed on chicks and hens [28–31]. Target species of rats were Sprague-Dawley in 10 studies [32–41], C57BL/6J mice in 9 studies [42–50], Wistar rat in 5 studies [51–55], and BALB/c mice in 5 studies [56–59]. The other studies used senescence-accelerated mouse (SAMP) [60, 61], virgin fisher rat [62], and ND4 Swiss Webster retired breeder mice [63]. Out of 37 studies, 17 publications were performed on male [29, 33, 35, 38, 41, 45, 46, 48, 49, 52–55, 60,61, 63, 64], 17 on female [30, 32, 34, 36, 37, 39, 40, 42,44, 50, 51, 56, 57, 59, 62, 65], and 3 on both gender [28, 31, 43]. In fourteen investigations, female rats had ovariectomy surgery that induced osteoporosis [36, 37, 39, 40, 42, 44, 47, 51, 56, 57, 59, 62, 66], and in two studies, diabetic rats were included [38, 45]. Animals were fed by *L. reuteri* [30, 31, 40, 43, 45, 46, 56, 57, 64], *L. casei* [34, 38–40, 51, 63], *L. paracasei* [42, 44, 54, 65], *L. plantarum* [42, 47, 63, 65]*, L. acidophilus* [40, 55, 59, 67]*, B. bifidum* [40, 53, 55, 63], *B. longum* [37, 47, 52, 53], *B. subtilis* [28, 29, 55], *L. helveticus* [32, 37, 41], *L. bulgaricus* [33, 35, 63], *Entrococcos faecium* [30, 31, 55], and *L. rhamnosus* [48, 49, 64]. Other studies used *B. breve* [53, 63], *B. animalis* [30, 31], *Streptococcus thermophilus* [33, 35], *Pediococcus acidilactici* [30, 31], *Escherichia coli* [49, 64], *Lactococcus lactis* [60, 61], *Bacillus licheniformis* [28], *Clostridium butyrium* [29], *Bacillus coagulans* [40], and Pasteurized *Akkermansia muciniphila* [50]. The dosage and complete name and species of probiotics were reported in Table 2. The sample size varied from 1 [61] to 120 [30] in each group. The duration of intervention was between 9 days [33, 35] and 11 months [61]. Although probiotic feeding had increased calcium [39], phosphorus [42, 48], 25-OH-D [40, 50], PTH [33], osteocalcin (OC) [33, 36, 44, 50, 51], and alkaline phosphatase (ALP) [40] levels in some investigations, reduced levels of ALP [39], acid phosphatase (ACP) [65], urinary calcium [41], and phosphorus [48] were observed in others. These different findings might be due to different age, sex, estrogen status, duration of intervention, and sample sizes. In terms of BMD, increased BMD in different sites were reported in previous publications, total [34, 35, 44, 51, 55], tibia [12, 30–32, 35, 54, 59], femur [30, 31, 49, 59], and calcaneus [35]. An increase in BMC was also reported in five investigations [30, 31, 40, 43, 57]. Trabecular thickness [34, 39, 43–45, 50, 51, 56, 59], bone volume [32, 34, 36, 38, 39, 42–44, 49, 50, 57, 59, 65], tibia length [29], femur weight [38, 65], and bone phosphorus [28, 52] and bone calcium [52, 53] were also affected by probiotic feeding in animals. In eight publications, probiotic feeding showed no effects on bone parameters [37, 46, 47, 60–64].

### 3.2. Clinical Trials

Characteristics of seven clinical trials regarding the effects of probiotic consumption on bone health status presented in Table 2. These studies are published between 2004 and 2020. Three of the publications were performed in European countries [20, 21, 68], two in Asian countries [19, 23], and two in the USA and Canada [22, 69]. All studies had randomized study design except for two of them [22, 68]. Out of seven included clinical trials, three studies were conducted on healthy postmenopausal women [19, 21, 68], two on postmenopausal women with osteopenia [20, 23], one in hypercholesterolemic adults [69], and one in overweight and obese adults [22]. Sample sizes were varied from 10 to 66 in the intervention group and 10 to 61 in the control group. The dosage and complete name and species of probiotics are reported in Table 2. Supplements contained 1.5 ^*∗*^ 10^8^ to 5 ^*∗*^ 10^10^ CFU of probiotics per dose. Several types of probiotics were consumed: *L. helveticus* [21], *L. reuteri* [68, 69], *B. subtilis* [19], and combination of various species [20, 22, 23]. The duration of intervention was between 1 day [21] and 12 months [20, 68]. Increased calcium [21] and 25-OH-D [69] level, and decreased parathyroid hormone [21, 23, 70], collagen type 1 cross-linked C-telopeptide (CTX) [20, 23], and bone-specific alkaline phosphatase (BALP) [23] level were demonstrated in these publications. Also, increased total hip BMD [19], and reduced BMD loss in L2-L4 [20], femoral neck [20], trochanter [20], and tibia [68] were indicated in these investigations. Almost all of the publications had high-quality score according to the Cochrane quality assessment tool for RCTs, except for one of them [22]. This study assessed the effect of symbiotic and probiotic consumption and BMC. Due to lack of effect sizes, we could not perform meta-analyses in BMC. Therefore, all high-quality score studies were included in different meta-analyses.

### 3.3. Meta-Analysis

**Figure 2:**
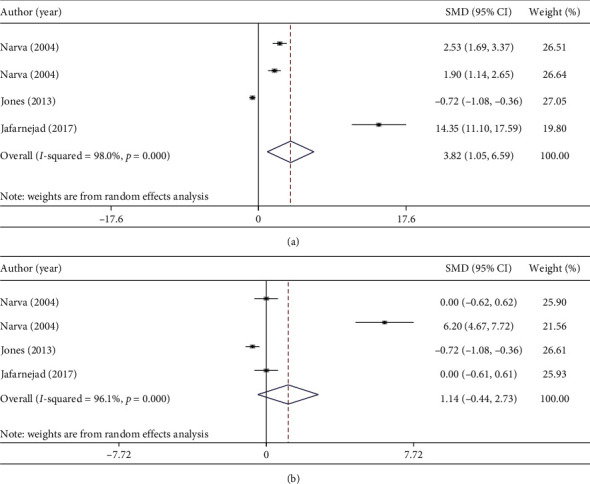
Forest plots for the effect of probiotic consumption on (a) serum calcium levels and (b) serum phosphorus levels, expressed as mean differences between intervention and the control diets.

**Figure 3:**
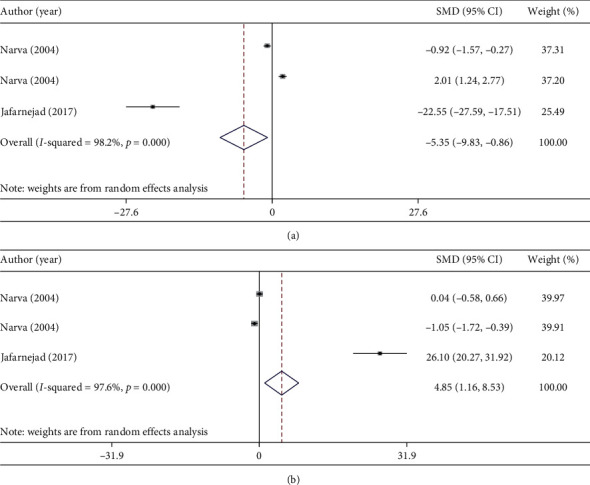
Forest plots for the effect of probiotic consumption on (a) PTH levels and (b) urinary calcium levels, expressed as mean differences between intervention and the control diets.

Some chemical parameters such as level of serum calcium, serum phosphorus, PTH, and urinary calcium had enough effect sizes (at least 3 effect sizes) to perform a meta-analysis to calculate combined results of probiotic consumption on bone health parameters. Most of clinical trials in this regard assessed the relation between probiotic consumption and bone health parameters in women 50 years and older. Combining four effect sizes of three studies indicated that probiotic consumption had significantly increased serum calcium levels (weighted mean difference (WMD): 3.82 mmol/l; 95% CI: 1.05, 6.59 mmol/l; *I*-square = 98.0%, *P* < 0.0001) (Figure 2(a)) [21, 23, 69]. Although significant heterogeneity was reported, low number of included studies did not let us to perform subgroup analysis and find source of heterogeneity. Combining four effect sizes of three studies, we did not find any significant effect of probiotic consumption on serum phosphorus levels (WMD: 1.14 mmol/l; 95% CI: −0.44, 2.73 mmol/l) (Figure 2(b)) [21, 23, 69]. In terms of PTH levels, probiotic consumption significantly decreases PTH levels (WMD: −5.53 ng/l; 95%CI: −9.83, −0.86 ng/l, *I*-square = 98.2%, *P* < 0.0001) (Figure 3(a)) [21, 23]. Combining three effect sizes of two studies, we found that probiotic consumption significantly influences urinary calcium levels (WMD: 4.85 mmol/l; 95% CI: 1.16, 8.53 mmol/l; *I*-square = 97.6%, *P* < 0.0001) (Figure 3(b)) [21, 23].

**Figure 4:**
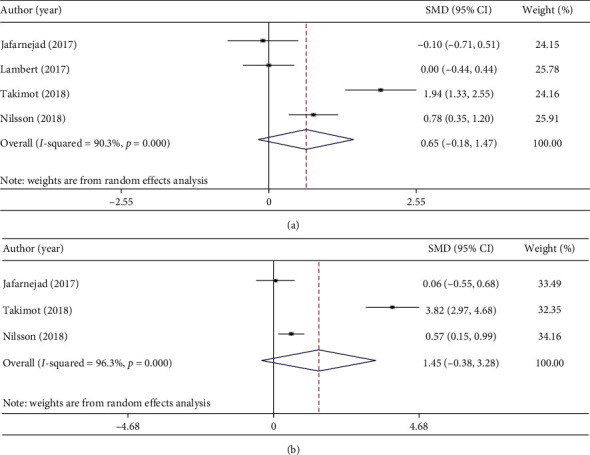
Forest plots for the effect of probiotic consumption on (a) spinal BMD levels and (b) total hip BMD levels, expressed as mean differences between intervention and the control diets.

Bone mineral density was calculated in different locations such as spinal, total hip, femoral neck, and troch. BMD at spinal and total hip had enough effect sizes, and we performed meta-analysis in these parameters. Combining four effect sizes of three studies indicated that probiotic consumption did not influence spinal BMD levels (WMD: 0.65 g/cm^2^; 95% CI: −0.18, 1.47 g/cm^2^) (Figure 4(a)) [19, 20, 23, 68]. In terms of total hip BMD, combining three effect sizes of three studies had shown nonsignificant increase in BMD level of total hip (WMD: 1.45 g/cm^2^; 95% CI: −0.38, 3.28 g/cm^2^) (Figure 4(b)) [19, 23, 68].

### 3.4. Sensitivity Analysis

To investigate the influence of each individual study on the overall findings, we excluded studies from the analysis, stage by stage, and found no significant impact of any individual study on the overall effect sizes.

### 3.5. Publication Bias

The funnel plots indicated moderate asymmetry, suggesting that publication bias cannot be completely excluded as a factor of influence on the present meta-analysis (data not shown). However, Begg's and Egger's regression tests provided no evidence of substantial publication bias.

## 4. Discussion

In this systematic review, we found some effects of probiotic supplementation on bone health parameters such as serum and urinary calcium levels and PTH levels. Some strains of *Bifidobacterium* and *Lactobacillus* such as *L. reuteri*, *L.* casei*, L. paracasei*, *L. bulgaricus*, and *L. acidophilus* indicated beneficial effects on bone health parameters in animal experiments and clinical trials.

Probiotic consumption has been assessed in few clinical trials. In most of them, probiotic consumption had beneficial effects on bone health parameters such as BMD, serum calcium, 25 (OH) D, and PTH levels. Only one clinical trial in this regard had reported no effect of probiotic consumption on bone health parameters that has been done on overweight and obese adults [22]. There are some critical points, which should be considered in the interpretation of the results of this study. This study had no randomization or blinding, so we cannot rule out the probable risk of bias. Notably, participants were obese or overweight, and we all know that this condition could change gut microbiota [71, 72] as well as hormonal status [70]. Considering all clinical trials, it seems that the consumption of probiotics may have positive effects on bone health in humans. Meta-analysis indicated that probiotic consumption improved some bone health parameters such as serum calcium levels and PTH. Significant heterogeneity is reported in our findings. Due to the small number of effect sizes, we could not use subgroup analyses to find the source of heterogeneity. Although studies in this area had high quality, they had some limitation. There are some differences between the previous included publications that could be the cause of heterogeneity. It seems that the mean age of the participants, gender, bone health status, and chronic conditions can justify the heterogeneity between studies.

Compared to clinical trials, the effects of probiotics on bone health have been addressed more in the animal experiments. Although in eight articles no effects of probiotics on bone health parameters were detected, most of the previous publications had reported beneficial effects of probiotic feeding on the bone health status of animals. There are several points that could explain the lack of connection between probiotic feeding and bone health parameters in these eight articles. Species of rats included in these surveys are different; virgin fisher rat [58], SAMP rat [60, 61], and ND4 Swiss Webster retired breeder mice [63] were used, while other studies used Sprague-Dawley rat, C57BL/6J mice, Wistar rat, and BALB/c mice. Sample sizes in one of these studies are very low (one rat in each group [60]), and older rats were included in these studies [37, 46, 47]. Considering the limitations of studies, it seems that probiotic feedings have beneficial effects on bone health parameters in animal experiments.

Gut microbiota is considered as an organ involving in mucosal barrier function, immune system, endocrine system, food digestion, and energy metabolism as well as bone health and metabolism [73–77]. Gut microbiota could regulate bone metabolism through the effects on the immune system, the endocrine organs, and calcium absorption. Some species of intestinal bacteria promote the release of inflammatory mediators, such as tumor necrosis factor-*α* (TNF *α*), interleukin (IL)-1, and IL-6 which plays an important role in the formation of osteoclasts and osteoblasts [78]. Intestinal microbiota also promotes the release of endothelial nitric oxide synthase (eNOS). eNOS mRNA regulates the production of osteoblasts and osteoclasts, as well as inflammatory mediators. NO has dichotomous biological effects, and at low concentrations, NO may promote proliferation, differentiation, and survival of osteoblasts, whereas at high concentrations, NO may inhibit bone resorption and formation. Therefore, at a certain concentration range, NO can avoid osteoclast-mediated bone resorption and promote osteoblast growth [79, 80]. Probiotics could affect gut microbiota and regulate immune cells and inflammatory cytokines or hormones and growth factors by inducing the host's production of *β*-defensin and IgA. Probiotics may also be able to enhance the intestinal barrier function by maintaining tight junctions and inducing mucin production. Probiotic-mediated immunomodulation may occur through the mediation of cytokine secretion signaling pathways such as NFκB and MAPKs, which plays a vital role in the formation of osteoclasts and osteoblasts [81–83]. Gut microbiota has also critical effects on the endocrine system. Levels of serum IGF-1 can promote the differentiation and growth of bone cells, including osteoblasts and chondrocytes, and enhance normal interactions among them [84]. Moreover, the IGF-1 signaling pathway is involved in the regulation of bone metabolism via both growth hormone (GH) and PTH which directly and indirectly have effects on bone growth [85]. PTH is secreted from parathyroid glands which regulate calcium levels by increasing absorption of calcium in gut, decreasing calcium absorption in kidney and increasing bone resorption. Bone remodeling is a dynamic coordination process between bone formation with osteoblasts and resorption with osteoclasts [86]. Increasing PTH level leads to more bone destruction by osteoclasts. As we indicated in meta-analysis results, greater calcium levels reduced PTH levels and osteoclast activity. Moreover, gonadal steroids, including estrogen and androgen, play key roles in the regulation of bone mass and turnover [87]. Gut microbiota regulates bone metabolism by affecting the absorption of calcium. Calcium absorption can be facilitated by vitamin D. It has been shown that a low-calcium diet alone can lead to bone resorption, high bone turnover, and impaired bone trabecular microarchitecture in multiple bones. Balanced gut microbiota leads to reduced osteoclast activity and increased osteoblast activity within the bone matrix by these strategies, which ultimately results in increased bone structure, density, and strength [88].

The present study has some strengths and limitations. It is the first study that systematically reviews the relationship between probiotic consumption and bone health parameters. In addition, a comprehensive search strategy was performed and no limitation was applied during the search. Furthermore, it is the first publication that performed meta-analysis on probiotic supplementations and bone health parameters. We considered experimental and clinical studies, and we tried to clarify the mechanism. However, some points need to be considered. Participants in the included clinical trials had different health status, for example, hyperlipidemia or obesity, which might influence the results. Moreover, different species and dosage of probiotics were used and it might impress findings. Although different probiotic species have different effects, due to the limited publications we combined findings of all studies. In addition, we could not perform meta-analyses in all parameters because of limited number of effect sizes. High heterogeneity was reported, and subgroup analyses could not be performed. Our findings might be considered as primary findings, and further studies should be designed on different bacterial species and strains.

## 5. Conclusion

In conclusion, in this systematic review, we found that probiotic supplementation containing *L. reuteri, L.* casei*, L. paracasei, L. bulgaricus, L. acidophilus*, and *B. subtilis* might improve bone health parameters in animal and human studies. Meta-analysis of human studies indicated that probiotic consumption has significantly increased serum and urinary calcium levels and decreased PTH level. Further studies are needed to decide on the appropriate probiotic species, strain, and dosages to improve bone health status.

## Figures and Tables

**Table 1:** Characteristics of studies that reported the relationship between probiotic consumption and bone health in animals.

Author (year)	Target species	Sample size (*n*)	Intervention group	Probiotic dose	Control group	Duration	Outcome	Results' mean ± SD (Int vs. ctrl)
Narva et al. (2004)	Six-week-old, male, Sprague-Dawley rats	5 groups (*n* = 10-11 per group)	1. *L. helveticus* (LBK-16H)-fermented milk + solid content of the same milk 2. *Saccharomyces*-fermented milk (*Saccharomyces cerevisiae* and *L. helveticus* bacteria)	1. 150 kJ/100 g 2. 200 kJ/100 g	1-sour milk fermented with a *Lactococcus* sp.-mixed culture 2-skim milk 3-water	14 weeks	U-Ca (mg/d)	Int1: 2.3 ± 0.5 Int2: 1.7 ± 0.3 Ctrl1: 0.6 ± 0.1 Ctrl2: 1.6 ± 0.2 Ctrl3: 0.7 ± 0.1
							BMC (g)	Int1: 0.451 ± 0.010 Int2: 0.409 ± 0.009 Ctrl1: 0.459 ± 0.008 Ctrl2: 0.474 ± 0.004 Ctrl3: 0.456 ± 0.008
							BMD (g/cm^2^)	Int1: 0.257 ± 0.018 Int2: 0.228 ± 0.003 Ctrl1: 0.243 ± 0.003 Ctrl2: 0.248 ± 0.002 Ctrl3: 0.241 ± 0.003
							Femur weight (g)	Int1: 0.89 ± 0.02 Int2: 0.82 ± 0.02 Ctrl1: 0.90 ± 0.02 Ctrl2: 0.92 ± 0.01 Ctrl3: 0.90 ± 0.01
							Femur volume (cm^3^)	Int1: 0.60 ± 0.01 Int2: 0.56 ± 0.01 Ctrl1: 0.61 ± 0.01 Ctrl2: 0.62 ± 0.01 Ctrl3: 0.62 ± 0.02
							Femur length (cm)	Int1: 3.64 ± 0.02 Int2: 3.55 ± 0.02 Ctrl1: 3.64 ± 0.02 Ctrl2: 3.68 ± 0.02 Ctrl3: 3.66 ± 0.03
							Ca (mg/g)	Int1: 162.0 ± 1.3 Int2: 160.9 ± 1.9 Ctrl1: 161.9 ± 1.4 Ctrl2: 164.7 ± 1.6 Ctrl3: 161.8 ± 1.8
							P (mg/g)	Int1: 74.8 ± 0.7 Int2: 74.6 ± 1.1 Ctrl1: 75.0 ± 0.8 Ctrl2: 76.4 ± 0.8 Ctrl3: 75.3 ± 1.1

Mutus et al. (2006)	1-day-old broiler chicks (both gender)	50 (*n* = 25 per each group)	Diet (corn, soybean meal, and wheat) + *Bacillus licheniformis* and *Bacillus subtilis*	Each containing 2.3 × 10^8^ CFU/g of spores)	Diet (corn, soybean meal, and wheat)	6 weeks	Tibiotarsi weight/length index (mg/mm)	79.59 vs. 77.22
							Robusticity index	4.94 vs. 5.01
							Diaphysis diameter (mm)	9.55 vs. 9.64
							Thickness of medial and lateral wall (mm)	1.75 vs. 1.58 3.03 vs. 2.57
							Medullary canal diameter (mm)	4.76 vs. 5.50
							Tibiotarsal index	49.99 vs. 43.26
							Modulus of elasticity (kg/cm^2^)	5192 vs. 4487
							Yield stress (kg/cm^2^)	83.48 vs. 81.56
							Bone-P (%)	11.26 vs. 10.06
							Bone-Ca (%)	23.63 vs. 22.52

Mathy et al. (2007)	90-day-old female Wistar rats	70, control: 10 rats (SH), intervention: 60 rats (OVX) (10 in each group)	1. Soy protein free powdered semipurified diet (control group) 2. Soy protein free powdered semipurified diet + genistein 3. Soy protein free powdered semipurified diet + daidzein 4. Soy protein free powdered semipurified diet + equol 5. Soy protein free powdered semipurified diet + daidzein + short chain FOS 6. Soy protein free powdered semipurified diet + daidzein + *Lactobacillus casei*	NM	Soybean-protein-free powdered semipurified diet	90 days	Total BMD (g/cm^2^)	OVX: 0.220 G: 0.234 D: 0.239 E: 0.239 D + FOS: 0.245 D + L: 0.241 SH: 0.256
							Diaphyseal BMD (g/cm^2^)	OVX: 0.209 G: 0.221 D: 0.225 E: 0.224 D + FOS: 0.230 D + L: 0.226 SH: 0.232
							Metaphyseal BMD (g/cm^2^)	OVX: 0.222 G: 0.246 D: 0.248 E: 0.251 D + FOS: 0.260 D + L: 0.255 SH: 0.267
							Femoral failure load	OVX: 102.67 G: 110.38 D: 109.18 E: 116.44 D + FOS: 115.02 D + L: 109.63 SH: 108.06
							OC (ng/ml)	OVX: 4.98 G: 5.45 D: 5.00 E: 4.89 D + FOS: 5.21 D + L: 5.12 SH: 3.91
							Trabecular thickness (um)	OVX: 9.200 G: 9.637 D: 9.883 E:9.050 D + FOS: 9.891 D + L: 10.899 SH: 9.090

Narva et al. (2007)	3-month-old female Sprague-Dawley rats	50 (10 in each group)	1. Tap water 2. 30 mg/L synthesized VPP peptide in water 3. 60 mg/L synthesized VPP peptide in water 4. *Lactobacillus helveticus*-fermented milk containing VPP peptide 21 mg/L	60 ml	Shamoperated group receiving tap water (sham)	12 weeks	Trabecular BMD of proximal tibia (mg/cm^3^)	Sham: 245.75 OVX: 105.25 OVX + VPP30 : 112.15 OVX + VPP60 : 108.30 OVX + LH: 126.61
							Cortical BMD of proximal tibia (mg/cm^3^)	Sham: 1271 OVX: 1255 OVX + VPP30 : 1256 OVX + VPP60 : 1260 OVX + LH:1266
							Cortical BMD of tibial diaphysis (mg/cm^3^)	Sham: 1367.2 OVX: 1364.6 OVX + VPP30 : 1367.7 OVX + VPP60 : 1367.6 OVX + LH: 1369.1
							Bone volume per tissue volume (%)	Sham: 20.43 OVX: 3.73 OVX + VPP30: 5.31 OVX + VPP60: 2.83 OVX + LH:6.53
							Trabecular thickness (um)	Sham: 46.52 OVX: 44.00 OVX + VPP30: 45.67 OVX + VPP60: 38.31 OVX + LH: 46.65

Kimoto-Nira et al. (2007)	SAMP male, 6 months	2 (*n* = 1 in each group)	Cornmeal + wheat + fishmeal + soya oil + calcium carbonate + vitamin/mineral (control) + strain H61	*Lactococcus lactis* subsp. *cremoris* H61 (strain H61)	Control diet	8 months	Bone density (mg/cm^2^)	44.42 vs. 38.49

Kimoto-Nira et al. (2009)	Male, 1-month-old, SAMP6 mice	Eighteen per experimental group	Cornmeal + wheat + fishmeal + soya oil + calcium carbonate + vitamin/mineral (control) + strain H61	*Lactococcus lactis* G50	Control diet	11 months	Bone density (mg/cm^2^)	39.7 ± 0.88 vs. 38.7 ± 0.88

Houshmand et al. (2010)	1-day male broiler chicks	150 (6 groups, *n* = 25)	1. Low-calcium diet (0.67%) 2. Low-calcium diet + probiotic 3. Low-calcium diet + prebiotic 4. Low-calcium diet + synbiotic 5. Low-calcium diet + organic acids (formic acid, citric acid, malic acid, lactic acid, tartaric acid, and orthophosphoric acid)	1 × 10^10^ CFU/kg *Bacillus subtilis* and 1 × 10^6^ CFU/kg *Clostridium butyricum*	Basal diet containing recommended level of calcium (0.9%)	21 days	TD incidence	Not detected in all groups
							Tibia length (cm)	Crtl: 6.87 ± 0.05 LC: 6.65 ± 0.04 LC + Pro:7.07 ± 0.09 LC + Pre: 6.92 ± 0.05 LC + Syn: 6.92 ± 0.05 LC + OA: 6.84 ± 0.06
							Tibia weight (g)	Crtl: 2.65 ± 0.13 LC: 2.18 ± 0.04 LC + Pro: 2.80 ± 0.14 LC + Pre: 2.82 ± 0.11 LC + Syn: 2.77 ± 0.11 LC + OA: 2.65 ± 0.08
							Strength (kg/m^2^)	Crtl:36.06 ± 2.36 LC: 33.35 ± 1.62 LC + Pro: 36.20 ± 2.10 LC + Pre: 35.84 ± 1.47 LC + Syn: 36.94 ± 3.12 LC + OA: 34.77 ± 1.49

Chiang and Pan (2011)	Female 2-month-old C57BL/6J mice	48 (40 mice were OVX, and 8 SH mice)	1. Phosphate-buffered saline (OVX) 2. Fosamax, 0.2 mg of alendronic acid, 8 units of colecalciferol per week (FOS) 3. 0.1 g of freeze-dried powder of soy skim milk fermented *by L. paracasei* subsp*. paracasei* NTU 101 (NTU 101F) 4. 0.1 g of freeze-dried powder of soy skim milk fermented by *L. plantarum* NTU 102 (NTU 102F) 5. 0.1 g of freeze-dried powder of nonfermented soy skim milk (NFSM)	(*Lactobacillus paracasei* subsp. *paracasei* NTU 101 and *Lactobacillus plantarum* NTU 102) 1.0 × 10^8^ CFU/mL	Phosphate-buffered saline (*n* = 8, sham)	8 weeks	Ca (mg/dl)	SH: 11.7 ± 0.3 OVX: 11.3 ± 0.4 FOS: 11.3 ± 0.3 NUT101F: 11.2 ± 0.4 NUT102F: 11.2 ± 0.3 NFSM: 11.1 ± 0.3
							P (mg/dl)	SH: 14.3 ± 1.5 OVX: 12.8 ± 1.2 FOS: 11.2 ± 0.8 NUT101F: 0.9 ± 0.7 NUT102F: 7.7 ± 1.1 NFSM: 7.7 ± 1.0
							ALP (U/L)	SH: 104.7 ± 14.6 OVX: 91.7 ± 11.1 FOS: 57.2 ± 15.5 NUT101F: 85.9 ± 9.0 NUT102F: 86.4 ± 11.5 NFSM: 96.3 ± 27.2
							ACP (U/L)	SH: 8.23 ± 0.55 OVX: 7.56 ± 0.67 FOS: 7.70 ± 0.71 NUT101F: 7.83 ± 0.75 NUT102F: 7.43 ± 0.86 NFSM: 7.01 ± 1.35
							BMD (g/cm^2^)	SH: 0.67 OVX: 0.66 FOS: 0.645 NUT101F: 0.63 NUT102F: 0.65 NFSM: 0.64
							Trabecular bone volume (%)	SH: 3.16 OVX: 2.00 FOS: 2.76 NUT101F: 3.08 NUT102F: 2.90 NFSM: 2.74
							Trabecular thickness (mm)	SH: 0.0719 OVX: 0.7266 FOS: 0.6727 NUT101F: 0.0716 NUT102F: 0.0706 NFSM: 0.0701

Chiang et al. (2011)	Female BALB/c mice, 3 months old	20 each group (*n* = 5)	1. 0.05 g freeze-dried powder of soy skim milk fermented by *L. plantarum* NTU 101 (NTU 101F) 2. 0.05 g freeze-dried powder of soy skim milk fermented by *L. plantarum* NTU 102 (NTU 102F) 3. 0.05 g freeze-dried powder of nonfermented soy skim milk (NFSM)	*Lactobacillus paracasei* subsp. *paracasei* strain NTU 101F (3.0 × 10^11^ CFU/g) & NTU 102F (3.9 × 10^11^ CFU/g)	Phosphate-buffered saline (PBS; control, ctrl)	10 months	Right femur weight (%)	Ctrl: 0.6 ± 0.0 NUT101F: 0.7 ± 0.0 NUT102F: 0.6 ± 0.1 NFSM: 0.7 ± 0.0
							Right femur length (cm)	Ctrl: 1.5 ± 0.1 NUT101F: 1.6 ± 0.0 NUT102F: 1.6 ± 0.1 NFSM: 1.6 ± 0.1
							Ca (mg/dl)	Ctrl: 9.8 ± 0.5 NUT101 F: 9.8 ± 0.3 NUT102F: 9.5 ± 0.3 NFSM: 10.2 ± 0.5
							P (mg/dl)	Ctrl: 7.0 ± 0.7 NUT101F: 7.0 ± 0.9 NUT102F: 7.3 ± 0.7 NFSM: 8.8 ± 1.5
							ALP (U/L)	Ctrl: 78.7 ± 8.2 NUT101F: 74.2 ± 4.6 NUT102F: 69.2 ± 9.3 NFSM: 70.6 ± 4.6
							ACP (U/L)	Ctrl: 5.2 ± 0.4 NUT101F: 3.9 ± 1.1 NUT102F: 3.6 ± 5.7 NFSM: 5.6 ± 1.3
							BMD (g/cm^2^)	Ctrl: 0.715 NUT101F: 0.723 NUT102F: 0.719 NFSM: 0.716
							Trabecular bone volume (%)	Ctrl: 1.08 NUT101F: 3.64 NUT102F: 2.38 NFSM: 1.95
							Trabecular thickness (mm)	Ctrl: 0.0835 NUT101F: 0.0798 NUT102F: 0.0776 NFSM: 0.735

Takasugi et al. (2011)	Male rats, aged 3 weeks, Sprague–Dawley rats	32 (*n* = 8 in each group)	1. DFL 2. DFL + PPI	Dairy product fermented by *Lactobacillus bulgaricus* and *Streptococcus thermophilus* (DFL)	1. Casein-based diet 2. Casein-based diet + PPI	9 days	Cortical BMD (mg/cm^3^)	Ctrl: 559 ± 12 Ctrl + PPI: 492 ± 9 DFL: 556 ± 8 DFL + PPI: 533 ± 9
							Cancellous BMD (mg/cm^3^)	Ctrl: 250 ± 7 Ctrl + PPI: 200 ± 5 DFL: 251 ± 8 DFL + PPI: 237 ± 6
							Total BMD (mg/cm^3^)	Ctrl: 357 ± 9 Ctrl + PPI: 303 ± 6 DFL: 359 ± 8 DFL + PPI: 341 ± 7
							Ca-left femur (mg)	Ctrl: 24.6 ± 0.9 Ctrl + PPI: 19.7 ± 0.6 DFL: 23.8 ± 0.7 DFL + PPI:22.5 ± 0.5
							P-left femur(mg)	Ctrl: 13.2 ± 0.5 Ctrl + PPI: 10.6 ± 0.3 DFL: 12.7 ± 0.4 DFL + PPI: 12.1 ± 0.3
							Total Ca (ug/ml)	Ctrl: 108 ± 2 Ctrl + PPI: 105 ± 2 DFL: 109 ± 3 DFL + PPI: 107 ± 2
							Total P (ug/ml)	Ctrl: 143 ± 9 Ctrl + PPI: 152 ± 6 DFL: 150 ± 6 DFL + PPI: 156 ± 8
							OC (ng/ml)	Ctrl: 756 ± 47 Ctrl + PPI: 965 ± 60 DFL: 755 ± 26 DFL + PPI: 784 ± 53
							CTX (ng/ml)	Ctrl: 74.8 ± 3.0 Ctrl + PPI: 92.1 ± 6.0 DFL: 77.8 ± 3.7 DFL + PPI: 78.3 ± 2.9
							PTH (pg/ml)	Ctrl: 61 ± 21 Ctrl + PPI: 215 ± 28 DFL: 73 ± 16 DFL + PPI: 163 ± 40
							25-OH-D (nmol/l)	Ctrl: 34.5 ± 1.8 Ctrl + PPI: 33.3 ± 2.2 DFL: 36.4 ± 1.7 DFL + PPI: 33.8 ± 2.2

Rodrigues et al. (2012)	Male Wistar rats, (aged = NM)	32 (*n* = 8 in each group)	1. Yacon flour (Y) 2. Diet + *B. longum* (B) 3. Yacon flour + *B. longum* (YB)	*Bifidobacterium longum*; 10^9^ CFU mL^−1^	1. Ctrl: AIN-93G diet	28 days	ALP (UI)	Ctrl: 156.88 ± 46.83 Y: 142.75 ± 25.89 B: 148.00 ± 29.71 YB: 139.12 ± 32.63
							Femur weight (g)	Ctrl: 0.60 ± 0.04 Y: 0.63 ± 0.04 B: 0.64 ± 0.04 YB: 0.64 ± 0.04
							Femur length (mm)	Ctrl: 3 2.30 ± 0.47 Y: 32.04 ± 0.87 B: 32.27 ± 0.59 YB: 31.40 ± 0.95
							Femur thickness (mm)	Ctrl: 4.60 ± 0.22 Y: 4.58 ± 0.32 B: 4.42 ± 0.25 YB: 4.50 ± 0.32
							Force of fracture (N)	Ctrl: 92.35 ± 11.19 Y: 99.86 ± 7.30 B: 100.33 ± 9.60 YB: 105.81 ± 21.70
							Bone weight (g)	Ctrl: 0.45 ± 0.03 Y: 0.47 ± 0.04 B: 0.45 ± 0.06 YB: 0.46 ± 0.02
							Bone-Ca	Ctrl: 7.55 ± 1.20 Y: 7.82 ± 1.07 B: 9.50 ± 0.57 YB: 8.91 ± 0.77
							Bone-P	Ctrl: 8.88 ± 1.21 Y: 9.18 ± 1.09 B: 10.75 ± 1.42 YB: 10.27 ± 0.57

Shim et al. (2012)	Sprague-Dawley female rats, 10 weeks	48 (sham, *n* *=* 8; OVX, *n* *=* 40)	1. Bilaterally OVX (OVX) 2. Bilaterally OVX + 0.3 g/kg of HRT (HRT-0.3) 3. Bilaterally OVX + 1.0 g/kg of HRT (HRT-1.0) 4. Bilaterally OVX followed by 0.3 g/kg of fHRT (fHRT-0.3) 5. Bilaterally OVX + 1.0 g/kg of fHRT (fHRT-1.0)	*Lactobacillus casei* KFRI-127 1–5 × 10^6^ CFU/mL	Sham operated (sham): normal diet without supplement	3 months	BMD (g/cm^3^)	Sham: 0.331 OVX: 0.094 HRT-0.3: 0.125 HRT-1: 0.095 fHRT-0.3: 0.107 fHRT-1: 0.276
							Bone volume (%)	Sham: 25.39 OVX: 8.98 HRT-0.3: 10.24 HRT-1: 7.94 fHRT-0.3: 8.96 fHRT-1: 18.68
							Trabecular thickness (mm)	Sham: 0.113 OVX: 0.098 HRT-0.3: 0.103 HRT-1: 0.105 fHRT-0.3: 0.103 fHRT-1: 0.124

McCabe et al. (2013)	C57Bl/6 mice, 14 weeks (both gender)	20 (*n* = 10 in each group)	Normal diet with supplement formula	*Lactobacillus reuteri* ATCC PTA 6475 (300 *μ*l of 1 × 109 CFU/ml)	Normal diet without supplement	4 weeks	Femur trabecular bone volume	28.8 ± 1.8 vs. 19.8 ± 1.7
							Femur trabecular thickness (mm)	49 ± 2 vs. 39 ± 2
							Femur trabecular BMC (mg)	0.58 ± 0.02 vs. 0.50 ± 0.02
							Femur trabecular BMD (mg/cc)	193 ± 7 vs. 166 ± 6
							Vertebral trabecular volume	56.9 ± 2.7 vs. 38.8 ± 2.7
							Vertebral trabecular thickness (mm)	61.3 ± 3.3 vs. 42.8 ± 1.7
							Vertebral trabecular BMC (mg)	0.61 ± 0.02 vs. 0.49 ± 0.02
							Vertebral trabecular BMD (mg/cc)	270 ± 7 vs. 216 ± 8

Takasugi et al. (2013)	3-week-old male Sprague–Dawley rats	56 (*n* = 8 in each group)	1. Normal (ctrl diet + vehicle) 2. Low GOL (LO) + PPI 3. High GOL (HO) + PPI 4. DFL + PPI 5. DFL + LO + PPI 6. DFL + HO + PPI	Dairy product fermented by *Lactobacillus bulgaricus* and *Streptococcus thermophilus* (DFL	1. Ctrl: modified AIN-93G diet + PPI	9 days	Cortical BMD (mg/cm^3^)	Normal: 518 ± 36 Ctrl: 475 ± 21 LO: 483 ± 21 HO: 515 ± 24 DFL: 542 ± 21 DFL + LO: 535 ± 13 DFL + HO:542 ± 30
							Cancellous BMD (mg/cm^3^)	Normal: 232 ± 14 Ctrl: 197 ± 12 LO: 203 ± 10 HO: 225 ± 18 DFL: 238 ± 13 DFL + LO: 236 ± 16 DFL + HO: 246 ± 20
							Total BMD (mg/cm^3^)	Normal: 330 ± 19 Ctrl: 294 ± 16 LO: 301 ± 13 HO: 327 ± 20 DFL: 345 ± 18 DFL + LO: 341 ± 15 DFL + HO: 349 ± 23

Britton et al. (2014)	BALB/c mice 12 weeks of age, non-OVX and OVX, female	24 (*n* = 8 in each group)	1. OVX with normal diet 2. OVX + *L. reuteri* with normal diet	300 *μ*l (1 × 10^9^ CFU/ml) *L. reuteri* ATCC PTA 6475 or MRS gavaging 1.5 × 10^8^ CFU/ml *L. reuteri* added water	Non-OVX (ctrl) with normal diet	Four weeks	Femur trabecular BMD (mg/cc)	Ctrl: 230 ± 1.2 OVX: 179 ± 7 OVX + Lr: 222 ± 11
							Femur trabecular BMC (mg)	Ctrl: 0.38 ± 0.02 OVX: 0.27 ± 0.01 OVX + Lr: 0.35 ± 0.02
							Femur trabecular thickness (um)	Ctrl: 45.4 ± 1.2 OVX: 35.8 ± 2.2 OVX + Lr: 41.3 ± 1.9
							Vertebrae trabecular BMD (mg/cc)	Ctrl: 218 ± 8 OVX: 182 ± 8 OVX + Lr: 204 ± 10
							Vertebrae trabecular BMC (mg)	Ctrl: 0.36 ± 0.01 OVX: 0.29 ± 0.01 OVX + Lr: 0.36 ± 0.01
							Vertebrae trabecular thickness (um)	Ctrl: 47.4 ± 1.81 OVX: 37.5 ± 1.00 OVX + Lr: 40.8 ± 1.48

Ohlsson et al. (2014)	Six-week-old C57BL/6N female mice	60 (*n* = 10 in each group)	1. OVX vehicle 2. OVX *L. para* 3. OVX *L. mix*	*Lactobacillus* strain, *L. paracasei* DSM13434 (*L. para*) or a mixture of three strains, *L. paracasei* DSM13434, *L. plantarum* DSM 15312 and DSM 15313 *supplementation*	1. Vehicle sham 2. *L. para* sham 3. *L. mix* sham	6 weeks	Trabecular bone volume (%)	Vehicle sham: 16.2 ± 0.7 *L. para* sham: 16.8 ± 0.8 *L. mix* sham: 17.4 ± 0.8 OVX vehicle: 13.2 ± 0.7 OVX *L. para*: 14.4 ± 0.6 OVX *L. mix*: 13.8 ± 0.5
							BMD (mg/cm^2^)	Vehicle sham: 322 ± 9 *L. para* sham:331 ± 12 *L. mix* sham:344 ± 8 OVX vehicle:285 ± 9 OVX *L. para*:302 ± 7 OVX *L. mix*:298 ± 7
							Trabecular thickness (mm)	Vehicle sham: 45.3 ± 0.7 *L. para* sham: 46.0 ± 0.7 *L. mix* sham: 47.9 ± 1.0 OVX vehicle: 43.2 ± 0.8 OVX *L. para*: 42.7 ± 0.8 OVX *L. mix*: 44.6 ± 1.0
							OC (ng/ml)	Vehicle sham: 90.9 ± 10.4 *L. para* sham: 97.1 ± 6.7 *L. mix* sham: 105.6 ± 6.1 OVX vehicle: 159.9 ± 11.8 OVX *L. para*: 142.1 ± 7.9 OVX *L. mix*: 136.9 ± 6.5
							Ca (mg/dl)	Vehicle sham: 9.1 ± 0.4 *L. para* sham: 9.2 ± 0.4 *L. mix* sham: 8.5 ± 0.3 OVX vehicle: 10.3 ± 0.4 OVX *L. para*: 9.3 ± 0.4 OVX *L. mix*:8.7 ± 0.3
							25 (OH) D3 (ng/ml)	Vehicle sham: 16.5 ± 1.3 *L. para* sham: 17.5 ± 1.7 *L. mix* sham: 16.5 ± 1.8 OVX vehicle: 16.7 ± 1.1 OVX *L. para*: 17.6 ± 1.0 OVX *L. mix*: 16.8 ± 1.2

Parvaneh et al. (2015)	10-week-old female mature Sprague-Dawley rats	24 (*n* = 8)	G2 : OVX, G3 : OVX + *Bifidobacterium longum* (OVX + *B. longum*).	1 mL of *Bifidobacterium longum* (108-109) (CFU/mL)	G1: sham-ovariectomized (sham)	16 weeks	Osteocalcin (ng/ml)	Sham: 184.61 ± 6.93 OVX: 76.81 ± 6.44 OVX + B: 101.31 ± 9.21
							Ca (mmol/L)	Sham: 2.27 ± 0.12 OVX: 2.23 ± 0.12 OVX + B: 2.27 ± 0.06
							Ca-femur (mg/g)	Sham: 246.23 ± 10.14 OVX: 242.09 ± 5.16 OVX + B: 242.45 ± 15.26
							Bone volume (%)	Sham: 73.66 ± 6.45 OVX: 49.60 ± 5.69 OVX + B: 60.55 ± 3.57
							Trabecular thickness (mm)	Sham:7.33 ± 0.65 OVX: 4.83 ± 0.27 OVX + B: 7.21 ± 0.48
							BMD (g/cm3)	Sham: 1.06 ± 0.02 OVX: 0.59 ± 0.07 OVX + B: 0.89 ± 0.06

Zhang et al. (2015)	Adult (14 weeks old) C57BL/6 male mice	40 (*n* = 10 in each group)	1. Normal diet + gavage *L. reuteri* (ctrl mice) 2. Normal diet + gavage *L. reuteri* (diabetic mice) 3. Normal diet (diabetic mice)	*Lactobacillus reuteri* ATCC PTA 6475 (10^9^ colony-forming units/mL)	Normal diet without gavage (ctrl mice)	4 weeks	BMD (mg/cc)	Ctrl: 223.05 D: 177.23 Ctrl + LR: 238.62 D + LR: 237.75
							BMC (mg)	Ctrl: 0.581 D: 0.502 Ctrl + LR: 0.634 D + LR: 0.657
							Trabecular thickness(um)	Ctrl: 42.59 D: 35.77 Ctrl + LR: 48.71 D + LR: 50.99
							Bone volume (%)	Ctrl: 31.23 D: 21.81 Ctrl + LR: 35.74 D + LR: 34.94

Collins et al. (2016)	Female BALB/c mice 11 weeks of age	4 group (*n* = 9–18 per group)	1. Nonsurgery (NS)+L. 2. Dorsal surgical incision (DSI)+L.	300 *μ*l (1 × 10^9^ CFU/ml) *Lactobacillus reuteri* 6475 or MRS	1. NS 2. DSI	4 or 8 week	Bone volume (%)	NS: 22.4 ± 2.1 DSI: 24.1 ± 1.7 NS + L: 20.9 ± 2.8 DSI + L: 31.4 ± 2.3
							Femur trabecular thickness (um)	NS: 48 ± 2 DSI: 45 ± 2 NS + L: 47 ± 3 DSI + L: 50 ± 2
							BMD (mg/cc)	NS: 857 ± 17 DSI: 905 ± 32 NS + L: 844 ± 10 DSI + L: 821 ± 32
							BMC(mg)	NS: 14.5 ± 0.5 DSI: 16.9 ± 0.5 NS + L: 14.7 ± 0.3 DSI + L.:16.2 ± 0.8

Scholz-Ahren et al. (2016)	Virgin female Fisher-344 rats 21-week age	80 (*n* = 15–16 in each group)	1. PRO : *L. acidophilus* NCC90 2. PRE: corn starch 2.5% 3. SYN : PRO + PRE	f 1–5 × 10^8^ CFU per 100 g	Sham OVX	16 weeks	Trabecular thickness (mm)	OVX: 0.020 ± 0.002 PRO: 0.020 ± 0.004 PRE: 0.020 ± 0.003 SYN: 0.021 ± 0.003
							U-Ca (mg/7 d)	OVX: 9.01 PRO: 8.70 PRE: 10.42 SYN: 10.82
							Ca-femur (mg)	OVX: 8.41 PRO: 8.64 PRE: 8.56 SYN: 8.78
							Ca-lumber vertebra (mg)	OVX: 41.14 PRO: 41.64 PRE: 43.35 SYN: 44.71
							U-P (mg/7 d)	OVX: 9.07 PRO: 8.93 PRE: 8.49 SYN: 8.34
							P-femur (mg)	OVX: 39.23 PRO: 40.25 PRE: 39.64 SYN: 4046
							P-lumber vertebra (mg)	OVX:19.38 PRO: 21.20 PRE: 21.12 SYN:21.56
							Bone alkaline phosphatase (BAP) (U/L)	OVX: 154.61 PRO: 158.85 PRE: 137.12 SYN: 126.93

Bayat et al. (2018)	Male Sprague-Dawley rats (aged = NM)	65 (*n* = 13 in each group)	1. Diabetic group (STZ): they were fed 1 ml/day of distilled water 2. Soy milk group (STZ + SM): diabetic rats were fed 1 ml/day of soy milk 3. Probiotic soy milk group (STZ + PSM): diabetic rats were fed 1 ml/day of probiotic soy milk 4. Omega-3 probiotic soy milk group (STZ + OPSM): diabetic rats were fed 1 ml/day of omega-3-enriched probiotic soy milk	*Lactobacillus casei* (1.5 × 10^8^ CFU/mL)	Nondiabetic group (control): they were fed 1 ml/day of distilled water	2 months	Tibia weight (mg)	Ctrl: 405 ± 14 D: 229 ± 29 SM: 310 ± 75 PSM: 312 ± 70 OPSM: 334 ± 42
							Tibia volume (mm^3^)	Ctrl: 243 ± 18 D: 124 ± 20 SM: 178 ± 56 PSM: 179 ± 39 OPSM: 191 ± 30
							Tibia trabeculae volume (mm^3^)	Ctrl: 119 ± 23 D: 57 ± 31 SM: 72 ± 25 PSM: 80 ± 26 OPSM: 88 ± 13
							Vertebra weight (mg)	Ctrl: 318 ± 34 D: 239 ± 50 SM: 275 ± 31 PSM: 293 ± 29 OPSM: 311 ± 41
							Vertebra volume (mg)	Ctrl: 207 ± 42 D: 116 ± 22 SM: 154 ± 49 PSM: 155 ± 37 OPSM: 182 ± 37
							Vertebra trabeculae volume (mm^3^)	Ctrl:8 6 ± 23 D: 45 ± 10 SM: 50 ± 25 PSM: 56 ± 14 OPSM: 60 ± 17

Blanton et al. (2018)	Male 8-month-old ND4 Swiss Webster retired breeder mice	18	1–10% grape powder 2–20% grape powder 3–10% grape powder + 1% probiotic 4–20% grape powder + 1% probiotic 5-1% probiotic	*Bifidobacterium bifidum*, *B. breve, Lactobacillus casei, L. plantarum,* and *L. bulgaricus* at a concentration of 1.0 × 10^11^ CFU/g	20% sugar	6 months	Tibia bone volume (%)	10%G: 5.9 ± 3.7 20%G: 6.4 ± 3.3 10%G + 1%P: 6.1 ± 2.2 20%G + %P: 3.67 ± 1.8 1%P: 6.1 ± 1.8 Ctrl: 6.4 ± 2.7
							Tibia thickness (mm)	10%G: 0.0392 ± 0.007 20%G: 0.042 ± 0.007 10%G + 1%P:0.038 ± 0.003 20%G + %P: 0.041 ± 0.006 1%P: 0.041 ± 0.004 Ctrl:0.035 ± 0.004
							Femur bone volume (%)	10%G: 8.4 ± 3.4 20%G: 8.5 ± 3.8 10%G + 1%P: 7.6 ± 3.5 20%G + %P: 5.6 ± 1.7 1%P: 7.4 ± 2.6 Ctrl: 7.2 ± 3.7
							Femur thickness (mm)	10%G: 0.050 ± 0.006 20%G: 0.046 ± 0.003 10%G + 1%P: 0.046 ± 0.002 20%G + %P: 0.044 ± 0.004 1%P: 0.048 ± 0.009 Ctrl: 0.044 ± 0.003

Dar et al. (2018)	Female mice (BALB/c) of 8–10 weeks	30 (*n* = 10 in each group)	Group B: OVX Group C: OVX + LA	200 *μ*l (of 10^9^ CFU/ml *Lactobacillus acidophilus* (LA) ATCC 4356	Group A: no probiotic + shamoperated	6 weeks	BMD of LV5(mgHA/cm^3^)	Sham: 2.14 ± 0.07 OVX: 1.74 ± 0.20 OVX + LA: 2.8 ± 0.08
							BMD of femur trabecular (mgHA/cm^3^)	Sham: 3.23 ± 0.08 OVX: 1.77 ± 0.40 OVX + LA: 3.54 ± 0.24
							BMD of tibia trabecular (mgHA/cm^3^)	Sham: 3.62 ± 0.58 OVX: 2.77 ± 0.19 OVX + LA: 3.51 ± 0.24
							BMD of femur cortical (mgHA/cm^3^)	Sham: 0.91 ± 0.02 OVX: 1.02 ± 0.016 OVX + LA:1.12 ± 0.02
							BMD of tibia cortical (mgHA/cm^3^)	Sham: 0.99 ± 0.012 OVX: 1 ± 0.03 OVX + LA: 1.11 ± 0.03
							Bone volume of LV5 (%)	Sham: 23.5 ± 0.04 OVX: 16.53 ± 1.61 OVX + LA:21.83 ± 0.04
							Bone volume of femur trabecular (%)	Sham:30.71 ± 0.32 OVX:13.21 ± 3.21 OVX + LA: 25.41 ± 1.51
							Bone volume of tibia trabecular (%)	Sham: 26.67 ± 0.35 OVX: 20.11 ± 0.35 OVX + LA: 25.31 ± 0.24
							Thickness of LV5 (mm)	Sham: 1.65 ± 0.08 OVX: 0.64 ± 0.16 OVX + LA: 1.42 ± 0.07
							Thickness of femur trabecular (mm)	Sham: 1.73 ± 0.14 OVX: 0.74 ± 0.16 OVX + LA:1.45 ± 0.08
							Thickness of tibia trabecular (mm)	Sham: 1.88 ± 0.02 OVX: 1.10 ± 0.02 OVX + LA: 1.76 ± 0.03

Eaimworawuthikul et al. (2018)	Six-week-old male Wistar rats	48 (*n* = 6 in each group)	1. High fat diet + vehicle (HFV) 2. High fat diet + probiotic (HFPO) 3. High fat diet + prebiotic (HFPE) 4. High fat diet + symbiotic (HFS)	*Lactobacillus paracasei* HII01 1 × 10^8^ CFU/ml/day	1. Normal diet + vehicle (NDV) 2. Normal diet + probiotic (NDPO) 3. Normal diet + prebiotic (NDPE) 4. Normal diet + symbiotic (NDS)	24 weeks	Trabecular volumetric BMD (g/cm^3^)	NDV: 0.978 NDPO: 0.881 NDPE: 0.878 NDS: 0.872 HFV: 0.844 HFPO: 0.877 HFPE: 0.858 HFS: 0.835
							Bone volume (%)	NDV: 24.11 NDPO: 32.39 NDPE: 35.57 NDS: 34.56 HFV: 20.72 HFPO: 19.58 HFPE: 21.71 HFS: 22.14
							Trabecular thickness (mm)	NDV: 0.325 NDPO: 0.387 NDPE: 0.343 NDS: 0.399 HFV: 0.306 HFPO: 0.265 HFPE: 0.267 HFS: 0.327

Montazeri-Najafabady et al. (2018)	Adult female Sprague-Dawley rats (12–14 weeks old)	49 (*n* = 7 in each group)	1. OVX (normal diet) 2. OVX + *Lactobacillus acidophilus* 3. OVX + *Lactobacillus Casei* 4. OVX + *Bacillus coagulans* 5. OVX + *Bifidobacterium* 6. OVX + *Lactobacillus reuteri*	*Lactobacillus*, *Streptococcus*, *Bifidobacterium* 1.5 × 10^8^ CFU/mL	Ctrl: normal diet	4 weeks	1,25 (OH) 2 D3 (Pmol/ml)	Ctrl: 19.86 OVX: 12.81 LBA: 14.48 LBC: 18.65 BCO: 16.14 BB: 15.40 LBR: 16.34
							P (mg/dl)	Ctrl: 6.48 OVX: 5.36 LBA: 4.13 LBC: 4.57 BCO: 4.49 BB: 5.11 LBR: 5.25
							Ca (mg/dl)	Ctrl: 10.06 OVX: 9.43 LBA: 9.77 LBC: 10.16 BCO: 9.43 BB: 9.77 LBR: 9.38
							ALP (IU/L)	Ctrl: 330.43 OVX: 512.76 LBA: 705.87 LBC: 743.05 BCO: 554.45 BB: 580.84 LBR: 690.63
							Global BMC (g)	Ctrl: 11.29 OVX: 9.09 LBA: 10.93 LBC: 10.69 BCO: 9.94 BB: 9.91 LBR: 10.17
							Femur BMC (g)	Ctrl: 0.600 OVX: 0.326 LBA: 0.603 LBC: 0.547 BCO: 0.574 BB: 0.569 LBR: 0.554
							Spine BMC (g)	Ctrl: 0.756 OVX: 0.717 LBA: 0.781 LBC: 0.762 BCO: 0.762 BB: 0.722 LBR: 0.745
							Tibia BMC (g)	Ctrl: 0.513 OVX: 0.288 LBA: 0.321 LBC: 0.500 BCO: 0.348 BB: 0.304 LBR: 0.287

Parvaneh et al. (2018)	Mature Sprague-Dawley rats, aged 10 weeks, female	24 (*n* = 8 in each group)	G2: OVX G3: OVX + *L. helveticus*	*Lactobacillus helveticus* (ATCC 27558) 1 mL of 10^8^–10^9^ CFU of *L. helveticus* in phosphate buffer saline	G1: sham	16 weeks	Bone volume (%)	Sham: 76 ± 6.30 OVX: 49 ± 5.7 OVX + L: 57 ± 6.6
							Trabecular thickness (mm)	Sham: 7.6 ± 0.5 OVX: 5.3 ± 0.4 OVX + L: 5.8 ± 0.5
							BMD (g/cm^3^)	Sham: 1.07 ± 0.02 OVX: 0.76 ± 0.06 OVX + L: 0.91 ± 0.05

Yan et al. (2018)	1-day-old Ross 708 broiler chicks, both gender	360 (*n* = 120 in each group)	1. 0.5X: regular diet + 0.5 g/kg symbiotic 2. 1X: regular diet + 1 g/kg symbiotic	*Enterococcus faecium,* *Pediococcus acidilactici, Bifidobacterium animalis,* and *Lactobacillus reuteri*	Ctrl: regular diet	40 days	Tibia BMD (g/cm^3^)	Ctrl: 0.209 0.5X: 0.216 1X: 0.226
							Tibia BMC (g)	Ctrl: 2.82 0.5X: 3.02 1X: 3.30
							Femur BMD (g/cm^3^)	Ctrl: 0.180 0.5X: 0.185 1X: 0.199
							Femur BMC (g)	Ctrl: 1.80 0.5X: 1.92 1X: 2.21
							Humerus BMD (g/cm^3^)	Ctrl: 0.215 0.5X: 0.214 1X: 0.234
							Humerus BMC (g)	Ctrl: 1.60 0.5X: 1.62 1X: 1.87

Yan et al. (2018)	56-week-old White Leghorn laying hens of the Hy-Line W-36 strain	96 (*n* = 24 in each group)	1-0.5X = 0.5 g/kg (0.5 × 106 CFU/g) 2-1X: 1 g/kg (1.0 × 2 × 106 CFU/g) 3-2X: 2 g/kg (2.0 × 4 × 106 CFU/g)	*Enterococcus faecium,* *Pediococcus acidilactici, Bifidobacterium animalis*, and *Lactobacillus reuteri*	Ctrl: regular diet	7 weeks	Tibia BMD (g/cm^2^)	Ctrl: 0.1912 0.5X: 0.2018 1X: 0.1978 2X: 0.2034
							Femur BMD (g/cm^2^)	Ctrl: 0.1931 0.5X: 0.2048 1X: 0.2023 2X: 0.2100
							Humerus BMD (g/cm^2^)	Ctrl: 0.1069 0.5X: 0.1102 1X: 0.1124 2X: 0.1136
							Keel BMD (g/cm^2^)	Ctrl: 0.1109 0.5X: 0.1122 1X: 0.1164 2X: 0.1138
							Tibia BMC (g)	Ctrl: 2.25 0.5X: 2.35 1X: 2.35 2X: 2.36
							Femur BMC (g)	Ctrl: 1.71 0.5X: 1.83 1X: 1.83 2X:1.86
							Humerus BMC (g)	Ctrl: 1.04 0.5X: 1.07 1X: 1.10 2X: 1.15
							Keel BMC(g)	Ctrl: 0.69 0.5X: 0.71 1X: 0.73 2X: 0.72
							CTX (c-terminal telopeptide of type I collagen) (ng/ml)	Ctrl:1.17 0.5X:0.97 1X: 1.05 2X: 0.99

Achi et al. (2019)	Male Wistar rats, aged = NM	48 (*n* = 8 in each group)	1-*B. breve* NCIM 5671, 2-*B. longum* NCIM 5672, 3-*B. bifidum* NCIM 5697 4-arthritic 5-standard anti-inflammatory drug (piroxicam)	*Bifidobacteria* strains *B. breve* NCIM 5671, *B. longum* NCIM 5672 *B. bifidum* NCIM 5697	Ctrl: regular diet	15 days	Ca-bone (mg/g)	*B. breve*: 182.94 ± 21.28 *B. longum*: 160.86 ± 18.71 *B. bifidum*: 163.20 ± 7.76 Arthritic: 139.15 ± 18.49 Piroxicam: 165.43 ± 14.02 Ctrl: 204.85 ± 4.15
							K-bone (mg/g)	*B. breve*: 3.013 ± 0.60 *B. longum*: 2.575 ± 0.68 *B. bifidum*: 2.839 ± 0.83 Arthritic: 2.007 ± 0.44 Piroxicam: 2.071 ± 0.77 Ctrl: 3.143 ± 0.56

Collins et al. (2019)	Male mice (12 weeks of age) wild-type (C57BL/6) and Rag knockout (Rag1tm1Mm, C57BL/6 background)	42 (*n* = 10–11 in each group)	1-WT + LR 2-RK + LR	3.3 × 10^8^ CFU/ml of *Lactobacillus reuteri* ATCC PTA 6475	1-WT 2-RK	4 weeks	Femur trabecular bone volume (%)	WT: 2.28 ± 3.33 WT + LR: 44.45 ± 4.16 RK: 40.53 ± 2.23 RK + LR:46.05 ± 5.25
							Femur trabecular BMD (mg/ml)	WT: 243.5 ± 8.13 WT + LR: 282.5 ± 12.41 RK: 265.2 ± 6.27 RK + LR: 290.3 ± 18.44
							Femur trabecular BMC (mg)	WT: 0.46 ± 0.02 WT + LR: 0.52 ± 0.01 RK: 0.51 ± 0.01 RK + LR: 0.52 ± 0.02
							Femur trabecular thickness (mm)	WT: 0.04 ± 0.002 WT + LR: 0.06 ± 0.004 RK: 0.05 ± 0.002 RK + LR:0.07 ± 0.006
							Femur cortical BMD (mg/ml)	WT: 784.3 ± 13.43 WT + LR: 779.7 ± 7.17 RK: 784.1 ± 7.14 RK + LR: 800 ± 11.65
							Femur cortical BMC (mg)	WT: 0.01 ± 0.001 WT + LR: 0.02 ± 0.00 RK: 0.01 ± 0.00 RK + LR: 0.02 ± 0.00

Lee et al. (2019)	Eight-week-old female Sprague-Dawley (SD) rats	40 (*n* = 10 per group)	1. Shamoperated (sham), 2 OVX 17-beta-estradioltreated (E2), 3. 500 mg/kg LABE-treated OVX (LAB)	*Lactobacillus casei* (LAB) LAB extract (LABE)	OVX-control (OVX)	8 weeks	Alp (U/L)	Sham: 112.53 OVX: 161.00 E2: 62.95 LABE: 75.21
							Ca (uM)	Sham: 44.06 OVX: 17.28 E2: 39.98 LABE: 22.78
							BMD (%)	Sham: 71.14 OVX: 42.62 E2: 54.42 LABE: 52.13
							Bone volume (%)	Sham: 16.83 OVX: 4.28 E2: 8.50 LABE: 7.45
							Bone trabecular thickness (mm)	Sham: 0.105 OVX: 0.096 E2: 0.111 LABE: 0.114

Levi et al. (2019)	Male Wistar rats (*Rattus norvegicus albinus*), aged = NM	40 (*n* = 10 per group)	Group PROB (control + probiotic); group CSI (cigarette smoke inhalation) group CSI + PROB (CSI + probiotic)	*Lactobacillus acidophilus* (1 × 10^9^ CFU/kg) *Enterococcus faecium* (2.1 × 10^9^ CFU/kg) *Bacillus subtilis* (2.9 × 10^9^ CFU/kg) *Bifidobacterium bifidum* (2 × 10^9^ CFU/kg)	Group ctrl (control, without CSI and probiotic)	6 months	BMD (%)	Ctrl: 0.780 PROB: 0.901 CSI: 0.478 CSI + PROB: 0.713
							Bone mineral volume (%)	Ctrl: 79.74 PROB: 80.10 CSI: 72.10 CSI + PROB: 74.56

Liu et al. (2019)	Six-week-old male C57BL6/J mice		1. Sham group: normal saline; 2. LGG group: Tenofovir disoproxil fumarate (TDF) (43 mg/kg) and LGG	*Lactobacillus rhamnosus* GG (5 × 10^8^ CFU, ATCC)	1. TDF group (negative control): 2. ZOL (zoledronate) group (positive control)	8 weeks	Ca (mmol/L)	Sham: 2.02 LGG: 1.98 TDF: 1.97 ZOL: 1.87
							P (mmol/L)	Sham: 2.93 LGG: 3.36 TDF: 3.04 ZOL: 3.18
							Mandibular BMD (mg/cm^3^)	Sham: 1219 LGG: 1177 TDF: 1011 ZOL: 1286
							Mandibular BMC (mg)	Sham: 1.23 LGG: 1.16 TDF: 0.72 ZOL: 1.30
							Bone volume (%)	Sham: 58.85 LGG: 57.66 TDF: 44.92 ZOL: 64.18
							Trabecular thickness (mm)	Sham: 0.063 LGG: 0.064 TDF: 0.048 ZOL: 0.074

Liu et al. (2019)	Mice aged 6 weeks and male (C57BL6/J)	5 group (*n* = 10–12)	(a) Sham group: normal saline (NS) (b) LGG + TDF group: 0.86 mg TDF (43 mg/kg body weight) + 5 × 10^8^ CFU LGG (c) *E. coli* + TDF group: TDF (43 mg/kg body weight) + 5 × 10^8^ CFU *E. coli*	*LGG* (accession number 53103) *Escherichia coli* (accession number 25922)	(d) TDF group: TDF (43 mg/kg body weight) (negative control). (e) ZOL + TDF group: TDF (43 mg/kg body weight) + ZOL (100 *μ*g/kg as a positive control)	8 weeks	Femur BMD (mg/cm^3^)	Sham: 544.172 ± 46.588 LGG + TDF: 565.929 ± 57.65 *E. coli* + TDF: 415.57 ± 18.319 TDF: 421.582 ± 14.481 ZOL + TDF: 1231.783 ± 124.543
							Femur BMC (mg)	Sham: 0.739 ± 0.132 LGG + TDF: 0.868 ± 0.169 *E. coli* + TDF: 0.551 ± 0.555 TDF: 0.623 ± 0.056 ZOL + TDF: 2.387 ± 0.324
							Bone volume (%)	Sham: 25.407 ± 3.821 LGG + TDF: 27.872 ± 0.671 *E. coli* + TDF: 16.757 ± 2.158 TDF: 16.223 ± 0.822 ZOL + TDF: 94.028 ± 2.278
							Trabecular thickness (mm)	Sham: 0.045 ± 0.01 LGG + TDF: 0.044 ± 0.003 *E. coli* + TDF: 0.038 ± 0.003 TDF: 0.039 ± 0.001 ZOL + TDF: 0.194 ± 0.017

Schepper et al. (2019)	Eleven-week-old male BALB/c mice	88 (*n* = 10–18 per group)	1. ABX: antibiotic-treated (ampicillin 1.0 g/L and neomycin 0.5 g/L) 2. ABX + LR 3. ABX + LGG 4. ABX + EC 5. ABX + MDY (high-molecular-weight polymer)	300 mL of *L. reuteri* 6475 (LR), *Lactobacillus rhamnosus* (LGG), nonpathogenic *Escherichia coli* (EC; ATCC O6 : B1) (1 × 10^9^ CFU/mL)	Ctrl: normal diet	4 week	BMD (mg/cc)	Ctrl: 1019 ± 24.34 ABX: 1024 ± 18.5 ABX + LR: 1045 ± 39.05 ABX + LGG: 1044 ± 55.7 ABX + EC: 1043 ± 27.23 ABX + MDY: 1021 ± 27.02
							BMC (mg)	Ctrl: 0.022 ± 0.001 ABX: 0.021 ± 0.0005 ABX + LR: 0.022 ± 0.001 ABX + LGG: 0.022 ± 0.001 ABX + EC: 0.023 ± 0.0007 ABX + MDY: 0.021 ± 0.001
							Cortical thickness (mm)	Ctrl: 0.29 ± 0.01 ABX: 0.28 ± 0.003 ABX + LR: 0.29 ± 0.003 ABX + LGG: 0.29 ± 0.003 ABX + EC: 0.28 ± 0.005 ABX + MDY: 0.28 ± 0.005

Lawenius et al. (2019)	Twelve-week-old female C57BL/6 mice	(*n* = 8–12/group	Sham + pAkk OVX + pAkk	Pasteurized *Akkermansia muciniphila* (pAkk) (2 × 108 CFU/150 *µ*l)	Sham + Veh OVX + Veh	4 week	Femur length (mm)	Sham + Veh: 15.37 ± 0.15 Sham + pAkk: 15.19 ± 0.07 OVX + Veh: 15.61 ± 0.08 OVX + pAkk: 15.27 ± 0.13
							Femur cortical thickness (mm)	Sham + Veh:0.197 ± 0.002 Sham + pAkk:0.192 ± 0.002 OVX + Veh: 0.186 ± 0.003 OVX + pAkk: 0.184 ± 0.002
							Vertebra length (mm)	Sham + Veh: 3.02 ± 0.02 Sham + pAkk: 3.04 ± 0.02 OVX + Veh: 3.06 ± 0.01 OVX + pAkk: 3.06 ± 0.01
							Vertebra cortical thickness (mm)	Sham + Veh: 0.065 ± 0.001 Sham + pAkk: 0.060 ± 0.002 OVX + Veh: 0.056 ± 0.001 OVX + pAkk: 0.056 ± 0.001
							25 (OH) D3 (ng/ml)	Sham + Veh: 41.7 ± 1.2 Sham + pAkk: 38.1 ± 2.8 OVX + Veh: 44.5 ± 1.3 OVX + pAkk: 42.9 ± 1.0
							OC (ng/ml)	Sham + Veh: 96.8 ± 3.8 Sham + pAkk: 104.1 ± 8.6 OVX + Veh: 116.3 ± 8.8 OVX + pAkk: 119.4 ± 7.3
							BMD (mg/cm^2^)	Sham + Veh: 77.62 Sham + pAkk: 70.54 OVX + Veh: 70.24 OVX + pAkk: 67.98
							Bone volume (%)	Sham + Veh: 15.63 Sham + pAkk: 14.34 OVX + Veh: 14.03 OVX + pAkk: 13.50
							Trabecular thickness (mm)	Sham + Veh: 0.0478 Sham + pAkk: 0.0442 OVX + Veh: 0.459 OVX + pAkk: 0.0436
							PTH (pg/ml)	Sham + Veh: 276 Sham + pAkk: 235 OVX + Veh: 506 OVX + pAkk: 330
							Ca (mg/dl)	Sham + Veh: 9.24 Sham + pAkk: 9.44 OVX + Veh: 9.16 OVX + pAkk: 9.33
							U–Ca	Sham + Veh: 0.237 Sham + pAkk: 0.484 OVX + Veh: 0.169 OVX + pAkk: 0.230

Kim et al. (2019)	Female C57BL/6 mice (6 weeks old for GV-induced BV and 11 weeks old for ovariectomy-induced osteoporosis)	56 (*n* = 8 in each group)	1. SH: vehicle (1% maltose) 2. OVX: vehicle (1% maltose) 3. OLP: 1 × 10^9^ CFU LP 4. OBL: 1 × 10^9^ CFU BL 5. OMX: 1 × 10^9^ CFU LP + BL 6. OCP: 1 mg/kg beta-estradiol	1 × 10^9^ CFU *Lactobacillus plantarum* NK3 and *Bifidobacterium longum* NK49	Ctrl: vehicle (1% maltose)	2 weeks (6 days per week)	Femur weight (g)	Ctrl: 0.178 SH: 0.173 OVX: 0.130 OLP: 0.171 OBL: 0.176 OMX: 0.177 OCP: 0.175
							Ca (mmol/L)	Ctrl: 0.280 SH: 0.288 OVX: 0.178 OLP: 0.269 OBL: 0.304 OMX: 0.258 OCP: 0.285
							P (mmol/L)	Ctrl: 0.871 SH: 0.920 OVX: 0.681 OLP: 0.861 OBL: 0.862 OMX: 0.945 OCP: 0.836
							OC (pg/ml)	Ctrl: 0.448 SH: 0.472 OVX: 0.059 OLP: 0.469 OBL: 0.441 OMX: 0.570 OCP: 0.609

Tibiotarsal index = (diaphysis diameter − medullary canal diameter)/diaphysis diameter × 100 (Barnet and Nordin, 1960). Robusticity index = bone length/cube root of bone weight (Reisenfeld, 1972). Calcium (Ca), phosphorus (P), urinary calcium (U-Ca), parathyroid hormone (PTH), osteocalcin (OC), isoleycyl-prolyl-proline (IPP), valyl-prolyl-proline (VPP), collagen type 1 cross-linked C-telopeptide (CTX), 25-hydroxy vitamin D (25-OH-D), alkaline phosphatase (ALP), acid phosphatase (ACP), bone mineral density (BMD), bone mineral content (BMC), Hwangryun-haedok-tang (HRT), fermented HRT (fHRT), sham-operated (SH), ovariectomized (OVX), tibia dyschondroplasis (TD), senescence-accelerated mouse (SAMP), and dorsal surgical incision (DSI). ^*∗*^Final results in each group were reported in the Results column. ^*∗∗*^Results with red color are significant (*P* value <0.05).

**Table 2:** Characteristics of studies that reported the relationship between probiotic consumption and bone health in humans.

Author (year)	Country	Study design	Population sex and age (y)	Sample size (*n*)	Intervention group (strain of probiotics)	Probiotic dose	Control group	Duration	Outcome	Results mean ± SD (Int vs. ctrl)	Quality score
Narva et al. (2004)	Finland	Randomized double-blind crossover study	Postmenopausal women 50 to 78 y (65 y)	20	*Lactobacillus helveticus* LBK-16H bacteria/milk fermented 14.5 g/100 g IPP &VPP	Portion size 220 ml	Normal sour milk fermented with a *Lactococcus* sp. mixed culture (420 ml)	1 day Int 6 day washout 1 day Int	Ca (mmol/l)	0.09 ± 0.01 vs. 0.05 ± 0.02 (↑)	H
									P (mmol/l)	−0.09 ± 0.02 vs. −0.09 ± 0.02	
									iCa (mmol/l)	0.03 ± 0.005 vs. 0.03 ± 0.004	
									PTH (ng/l)	−20.8 ± 5.3 vs. −15.4 ± 6.4 (↓)	
									ICTP (ug/l)	−1.09 ± 0.28 vs. −1.15 ± 0.20	
									U-Ca (mmol/l)	0.19 ± 0.5 vs. 0.17 ± 0.5	
					Orange juice + peptide fraction from *L. helveticus* fermented milk whey with the same amount of IPP and VPP as in the first product	400 ml	Orange juice + calcium lactate gluconate (220 ml)	1 day Int 6 day washout 1 day Int	Ca (mmol/l)	0.10 ± 0.02 vs. 0.07 ± 0.01 (↓)	
									P (mmol/l)	−0.12 ± 0.02 vs. −0.11 ± 0.01	
									iCa (mmol/l)	0.005 ± 0.004 vs. 0.026 ± 0.005	
									PTH (ng/l)	−13.0 ± 4.7 vs. −23.7 ± 5.9 (↑)	
									ICTP (ug/l)	−1.10 ± 0.10 vs. −1.08 ± 0.18	
									U-Ca (mmol/l)	0.15 ± 0.04 vs. 0.21 ± 0.07 (↓)	

Jones et al. (2013)	Canada	Double-blind, placebo-controlled, randomized, parallel-arm study	Hypercholesterolemic adults 34–64 y (50 y)/both gender	127 (61 Pbo) (66 Int)	Capsule supplement/*Lactobacillus reuteri* NCIMB 30242	2 capsules (130 mg *L. reuteri* (2.9 × 10^9^) + 170 mg MD)	300 mg MD	13 week	Ca (umol/l)	−0.01 ± 0.001 vs. 0.01 ± 0.02	H
									P (umol/l)	−0.03 ± 0.001 vs. −0.01 ± 0.04	
									25 (OH) D (nmol/L)	14.73 ± 13.38 vs. −3.19 ± 5.63 (↑)	

Jafarnejad et al. (2017)	Iran	Randomized, double-blind, placebo-controlled study	Women with osteopenia 50–72 y (58 y)	41 (20 Int) (21 Pbo)	Multispecies probiotic capsules (GeriLact) (7 bacteria species: *Lactobacillus casei* 1.3 × 10^10^, *Bifidobacterium longum* 5 × 10^10^, *Lactobacillus acidophilus* 1.5 × 10^10^, *Lactobacillus rhamnosus* 3.5 × 10^9^, *Lactobacillus bulgaricus* 2.5 × 10^8^, *Bifidobacterium* breve 1 × 10^10^, and *Streptococcus thermophilus* 1.5 × 10^8^)	1 capsule 500 mg	Placebo capsule 500 mg of corn starch	6 months	Spinal BMD (g/cm^2^)	0.001 ± 0.01 vs. 0.002 ± 0.01	H
									Total hip BMD (g/cm^2^)	−0.015 ± 0.01 vs. −0.016 ± 0.02	
									BALP (U/L)	−3.12 ± 0.76 vs. 0.82 ± 0.06 (↓)	
									OC (ng/ml)	−0.16 ± 0.07 vs. −1.62 ± 0.2	
									CTX (ng/ml)	−0.06 ± 0.001 vs. −0.03 ± 0.001 (↓)	
									PTH (pg/ml)	−2.87 ± 0.14 vs. 2.16 ± 0.28 (↓)	
									ALP (U/L)	7.4 ± 0.7 vs. 7.5 ± 0.4	
									Ca (mg/dl)	0.75 ± 0.12 vs. −0.52 ± 0.04	
									P (mg/dl)	0.29 ± 0.04 vs. 0.05 ± 0.001	
									25 (OH) D (ng/ml)	28.20 ± 0.97 vs. 29.08 ± 1	
									U-Ca (mg/dl)	14.5 ± 0.5 vs. 3.8 ± 0.3	

Lambert et al. (2017)	Denmark	Double-blind, parallel design, placebo-controlled, randomized controlled trial	Postmenopausal osteopenic women 59–64 y (61 y)	78 (38 Int) (40 Pbo)	60 mg isoflavone aglycones and acid lactic probiotics, cold fermentation	2 Sachet 95 ml (RCE extract + probiotic)	2 Sachet 95 ml (water + food color)	12 month	CTX (ng/ml)	−0.05 ± 0.13 vs. 0.03 ± 0.16 (↓)	H
									P1NP (ng/ml)	1.56 ± 0.53 vs. 0.7 ± 0.56	
									OC (ng/ml)	−0.03 ± 0.29 vs. 0.69 ± 0.22	
									BMD changes L2–L4 (g/cm^2^)	−0.0085 (−0.017, 0.00006) vs. −0.022 (−20.032, 20.012) (↓)	
									BMD changes FN (g/cm^2^)	−0.008 (−0.015, 0.00003) vs. −0.022 (−0.03, −0.15) (↓)	
									BMD changes troch (g/cm^2^)	−0.004 (−0.01,0.004) vs. −0.017 (−0.025, −0.008) (↓)	

Takimoto et al. (2018)	Japan	Randomized, placebo-controlled, double-blind clinical trial	Healthy women 50-69 y (57 y)	61 (31 Int, 30 Pbo)	Tables contain soybean oil residue (0.34 mg) + *Bacillus subtilis* C-3102	0.19 g (3.4 × 10^9^)	0.19 g	26 weeks	BMD changes L2–L4 (g/cm^2^)	0.18 ± 0.50 vs. −0.68 ± 0.63	H
									BMD changes total hip (g/cm^2^)	2.53 ± 0.52 vs. 0.83 ± 0.35 (↑)	

Nilsson et al. (2018)	Sweden	Double-blind, placebo-controlled study	Women 75–80 y (76 y)	90 (45 Int, 45 Pbo)	Capsules contain *Lactobacillus reuteri* ATCCPTA 6475 + MD	2 capsules 5 × 10^9^	MD	12 months	Tibia total vBMD changes	−0.83 (−1.47, −0.19) vs. −1.85 (−2.64, −1.07) (↓)	H
									BMD changes L2–L4 (g/cm^2^)	0.78 (−0.54, 2.10) vs. 0.08 (−1.02, 1.19)	
									BMD changes total hip (g/cm^2^)	−0.13 (−1.33, 1.07) vs. −0.90 (−2.07, 0.27)	
									BAP (U/L)	−4.83 (−13.8, 13.1) vs. 5.43 (−12.8, 22.0)	

Sergeev et al. (2020)	The USA	Placebo-controlled clinical trial	Overweight and obese adults 31–62 y (47 y)/both gender	20 (10 Int, 10 Pbo)	Capsule (*Lactobacillus acidophilus* DDS‐1, *Bifidobacterium lactis* UABla‐12, *Bifidobacterium longum* UABl‐14, and *Bifidobacterium bifidum* UABb‐10) + a trans‐galactooligosaccharide (GOS)	69 mg (15 × 10^9^) 5.5 g	Placebo capsule	3 month	BMC changes (kg)	0.75 ± 0.05 vs. 0.16 ± 0.01	L

Intervention (Int), placebo (Pbo), calcium (Ca), phosphate (P), serum ionised calcium (iCa), parathyroid hormone (PTH), carboxyterminal telopeptide of type I collagen (ICTP), urinary calcium (U-Ca), isoleycyl-prolyl-proline (IPP), valyl-prolyl-proline (VPP), maltodextrin (MD), bone-specific alkaline phosphatase (BALP), osteocalcin (OC), control (ctrl), collagen type 1 cross-linked C-telopeptide (CTX), bone mineral density (BMD), bone mineral content (BMC), alkaline phosphatase (ALP), bone-specific alkaline phosphatase (BAP), femoral neck (FN), lumber spine (L), trochanter (Troch), procollagen type I *N*-terminal propeptide (P1NP), 25-hydroxy vitamin D (25-OH-D), volumetric bone mineral density (vBMD), red clover extract (RCE), year (y), and gram (g). ^*∗*^Results are presented with mean differences and standard deviation, and red color results are significant (*P*_value_ < 0.05).

## Data Availability

The (effect sizes) data used to support the findings of this study are included within the article. The (search strategy) data used to support the findings of this study are also included within the supplementary information file.

## Supplementary Materials

Supplementary Materials Supplementary table 1: search strategies and the number of publications in each electronic database.
