# Supplementary material for: Probiotics as a New Regulator for Bone Health: A Systematic Review and Meta-Analysis
Source: Evid Based Complement Alternat Med. 2021 Aug 2;2021:3582989. doi: 10.1155/2021/3582989 (PMC8355998; doi:10.1155/2021/3582989)
Supplement: Supplementary Materials — Supplementary table 1: search strategies and the number of publications in each electronic database. [file 3582989.f1.docx]

| Supplementary table 1. Search strategies and the number of publications in each electronic database | |
| --- | --- |
| Database | Search strategy |
| PubMed | (probiotic[MeSH Terms] OR probiotic[Title/Abstract] OR symbiotic [Title/Abstract] OR lactobacillus[Title/Abstract] OR lactobacillus[MeSH Terms] OR “bifid bacterium” [Title/Abstract]) **AND** ("fractures, bone"[MeSH Terms] OR "osteoporosis, postmenopausal"[MeSH Terms] OR "osteoporosis"[MeSH Terms] OR "alkaline phosphatase"[MeSH Terms] OR "osteocalcin"[MeSH Terms]  OR **osteoporosis[Title/Abstract] OR fracture[Title/Abstract] OR "bone mineral density"[Title/Abstract] OR BMD[Title/Abstract] OR "bone mineral content" [Title/Abstract] OR "alkaline phosphatase"[Title/Abstract] OR osteocalcin[Title/Abstract] OR "procollagen type 1 N-terminal propeptide"[Title/Abstract] OR hydroxyproline[Title/Abstract] OR "NF-kB ligand"[Title/Abstract])** |
| Scopus | ( ( TITLE-ABS-KEY ( bmd ) OR TITLE-ABS-KEY ( "bone mineral content" ) OR TITLE-ABS-KEY ( "alkaline phosphatase" ) OR TITLE-ABS-KEY ( "bone mineral density" ) OR TITLE-ABS-KEY ( fracture ) OR TITLE-ABS-KEY ( osteoporosis ) OR TITLE-ABS-KEY ( "procollagen type 1 N-terminal propeptide" ) OR TITLE-ABS-KEY ( hydroxyproline ) OR TITLE-ABS-KEY ( "NF-kB ligand" ) ) ) **AND** ( ( TITLE-ABS-KEY ( probiotic ) OR TITLE-ABS-KEY ( symbiotic ) OR TITLE-ABS-KEY ( lactobacillus ) OR TITLE-ABS-KEY ( "bifid bacterium" ) ) ) |
| EMBASE | ('osteoporosis'/exp OR osteoporosis OR 'fracture'/exp OR fracture OR 'bone mineral density'/exp OR 'bone mineral density' OR bmd OR 'bone mineral content'/exp OR 'bone mineral content' OR 'alkaline phosphatase'/exp OR 'alkaline phosphatase' OR 'osteocalcin'/exp OR osteocalcin OR 'procollagen type 1 n-terminal propeptide' OR 'hydroxyproline'/exp OR hydroxyproline OR 'nf-kb ligand')  **AND**('probiotic'/exp OR probiotic OR symbiotic OR 'lactobacillus'/exp OR lactobacillus OR 'bifid bacterium') |
| ISI Web of Science | TOPIC: (osteoporosis) *OR* TOPIC: (fracture) *OR* TOPIC: (“bone mineral density”) *OR* TOPIC: (“bone mineral content”) *OR* TOPIC: (BMD) *OR* TOPIC: (“alkaline phosphatase”) *OR* TOPIC: (osteocalcin) *OR* TOPIC: ("procollagen type 1 N-terminal propeptide") *OR* TOPIC: (hydroxyproline) *OR* TOPIC: ("NF-kB ligand") **AND** TOPIC: (probiotic) *OR* TOPIC: (symbiotic) *OR* TOPIC: (Lactobacillus) *OR* TOPIC: (Bifidobacterium) |
